# Understanding the Distinction Between Traumatic Fibroma and Mucocele in Pediatric Patients: A Report of Two Cases

**DOI:** 10.7759/cureus.55631

**Published:** 2024-03-06

**Authors:** Dhruvi Solanki, Punit Fulzele, Nilima R Thosar, Rutuja Ragit, Unnati Shirbhate, Ishani Rahate, Harikishan Kanani

**Affiliations:** 1 Department of Pedodontics and Preventive Dentistry, Sharad Pawar Dental College, Datta Meghe Institute of Higher Education & Research, Wardha, IND; 2 Department of Periodontics, Sharad Pawar Dental College, Datta Meghe Institute of Higher Education & Research, Wardha, IND

**Keywords:** lip biting, lip lesions, case report, pediatric oral lesions, traumatic lesions, salivary gland diseases, lip pathologies, laser, mucocele, traumatic fibroma

## Abstract

Traumatic fibroma is a reactive oral cavity lesion that manifests as a localized, non-neoplastic, inflammatory hyperplastic papule of fibrous connective tissue. Alternatively, mucocele is another frequent oral lesion, caused by mucus pooling in the tissues as a result of trauma to minor salivary glands. This article aims to shed light on traumatic fibroma and mucocele of the lower lip in pediatric patients. Two pediatric patients complained of soft tissue growth on the left side of the lower lip. Appropriate diagnosis, treatment planning, and light amplification by stimulated emission of radiation excision were done for both patients. The excised samples were sent for histopathological analysis. Both patients showed clinical resolution in a short period without any discomfort. A comprehensive understanding of these variances is essential for precise diagnosis and tailored treatment strategies.

## Introduction

Irritation fibroma, also known as traumatic fibroma, fibrous hyperplasia, or focal fibrous overgrowth, is a reactive oral cavity lesion that manifests as a localized, non-neoplastic, inflammatory hyperplastic papule of fibrous connective tissue. It is a rare benign tumor affecting pediatric patients and develops as a result of chronic irritation to the lip tissues. “Biting fibroma” arises from self-biting [1]. On the other hand, mucocele is the most frequent oral lesion, caused by mucus pooling in the tissues due to trauma to minor salivary glands. Histologically, they are characterized as extravasation and retention type. It is the 17th most common salivary gland lesion [2]. Traumatic fibroma and mucocele are two distinct oral lesions that can manifest in the same location, the lip. Although they share some similarities, they differ in their underlying causes, clinical presentations, and treatment approaches. Both are generally harmless but can cause discomfort, interfere with daily activities, and lead to aesthetic concerns. This article aims to shed light on traumatic fibroma as well as mucocele of the lower lip in pediatric patients by exploring its causes, symptoms, diagnosis, and appropriate management as well as the differences between them.

## Case presentation

Case one

An eight-year-old patient was brought to the Pediatric Dentistry Department by his father, with complaints of soft tissue growth on the left side of the child’s lower lip for 15 months. The growth was smaller initially and had increased in size gradually. It was not associated with pain, pus, or blood discharge. The patient was not on any medication, and no relevant medical and dental history was provided. The patient bit his lower lip as a habit, particularly when he was under stress.

**Figure 1:**
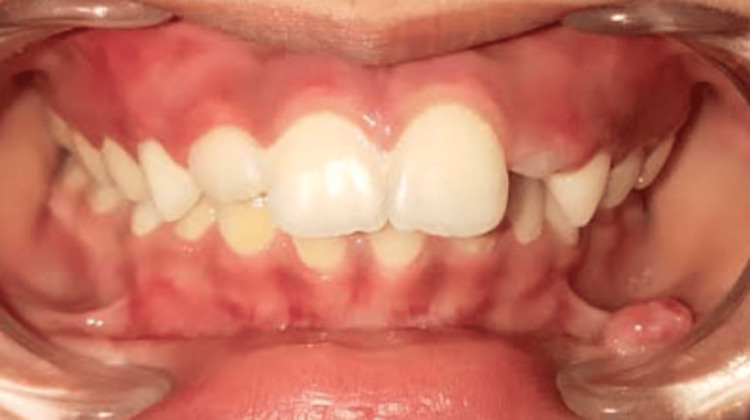
Intraoral image showing a sessile mass present on the lower left labial mucosa in relation to the 32 to 73 region.

**Figure 2:**
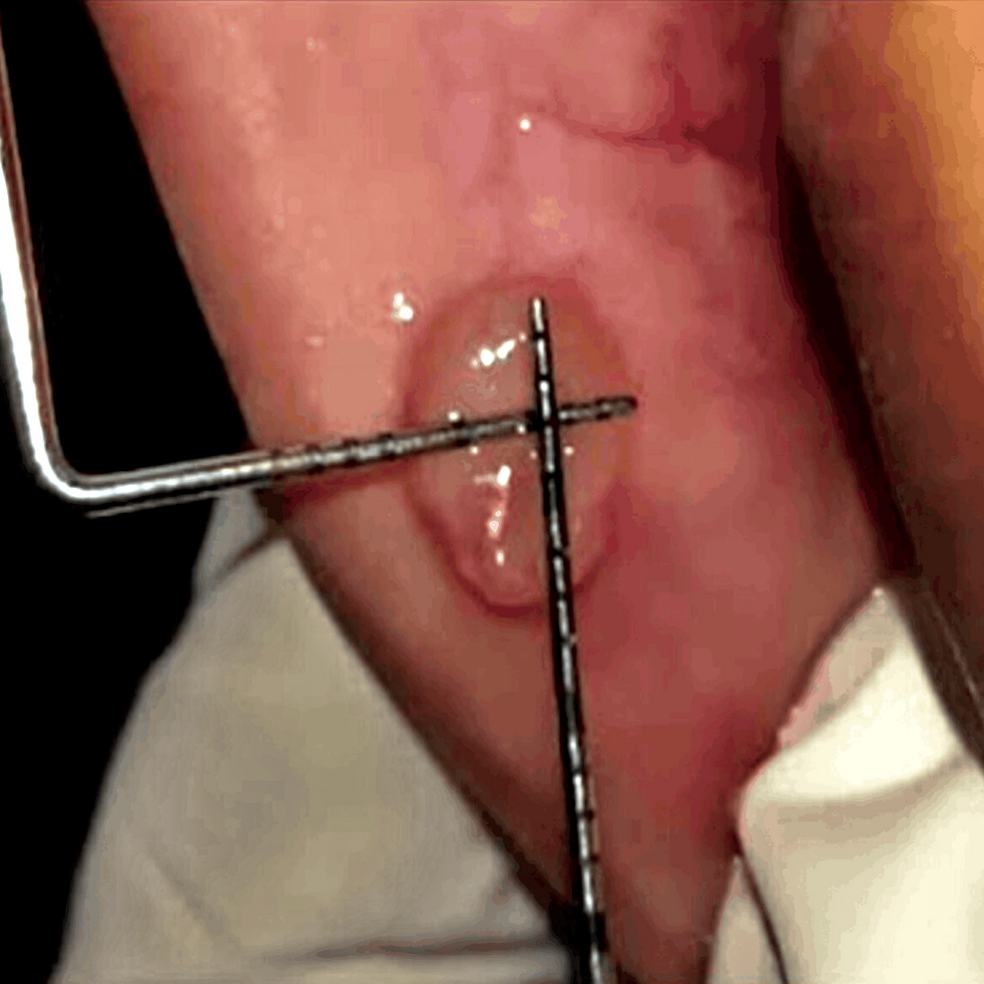
Mass on the lower lip measuring size approximately 0.7 x 0.5 cm.

On extraoral examination, no abnormality was detected and the lips were competent. Intraoral examination revealed a single sessile mass on the lower left labial mucosa in relation to the 32 to 73 region, measuring approximately 0.7 x 0.5 cm. The growth was ovoid-shaped with well-defined margins. The surface was glossy and the color was the same as the adjacent mucosa (Figures 1, 2).

On palpation, tenderness was negative and the consistency was soft to firm. Apart from the growth, tooth number 22 was found to be erupting. Based on the history and clinical findings, a provisional diagnosis of traumatic fibroma was given, with mucocele as the differential diagnosis.

**Figure 3:**
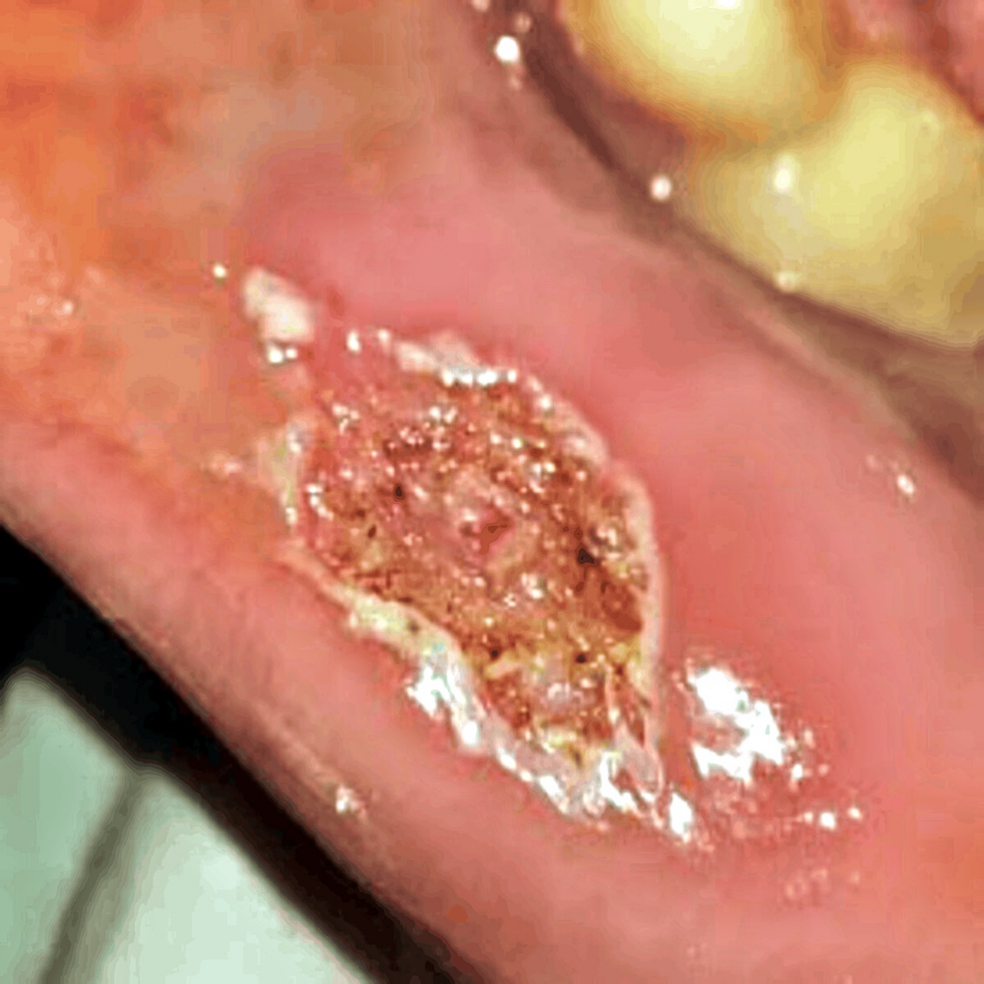
Immediate postoperative image after LASER excision.

Light amplification by stimulated emission of radiation (LASER) excision was planned to remove the fibroma, for which local anesthesia was first given at the borders of the tissue growth to anesthetize the same. Diode LASER was used in contact mode at 940 nm wavelength and 10 W (Figure 3).

**Figure 4:**
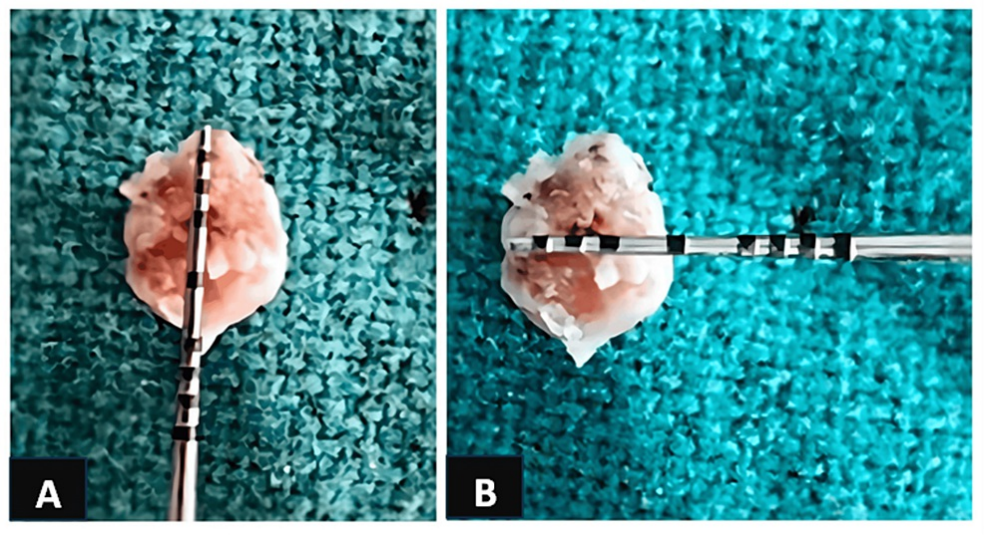
Excised tissue mass. A: Vertical measurement. B: Horizontal measurement.

Subsequently, the tissue sample was sent for histopathological examination for final diagnosis (Figure 4).

**Figure 5:**
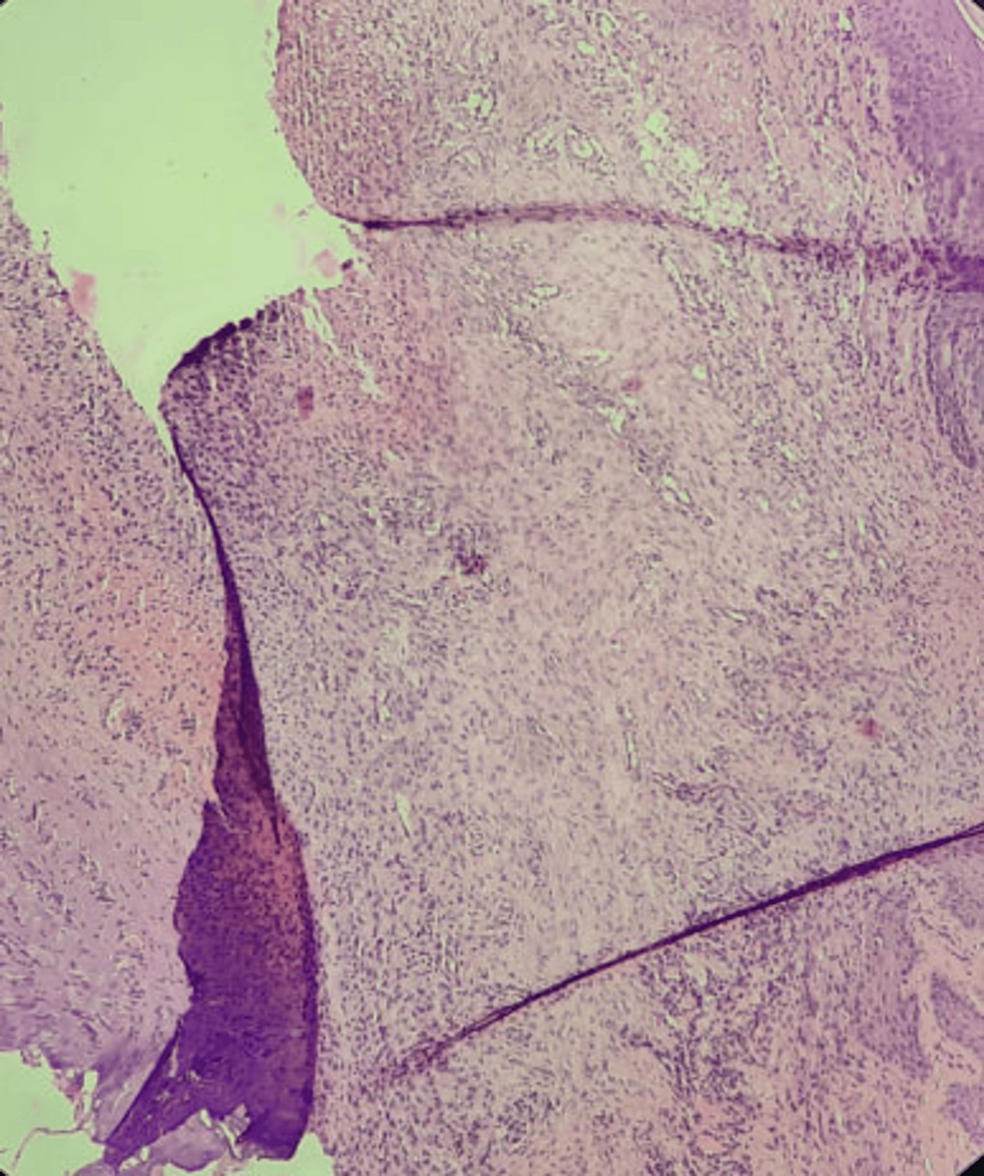
Histopathological image of the excised specimen stained with hematoxylin and eosin, showing connective tissue with numerous fibroblasts and collagen deposition at 10× magnification suggestive of fibroma.

The histopathological results were consistent with that of the clinical diagnosis (Figure 5).

**Figure 6:**
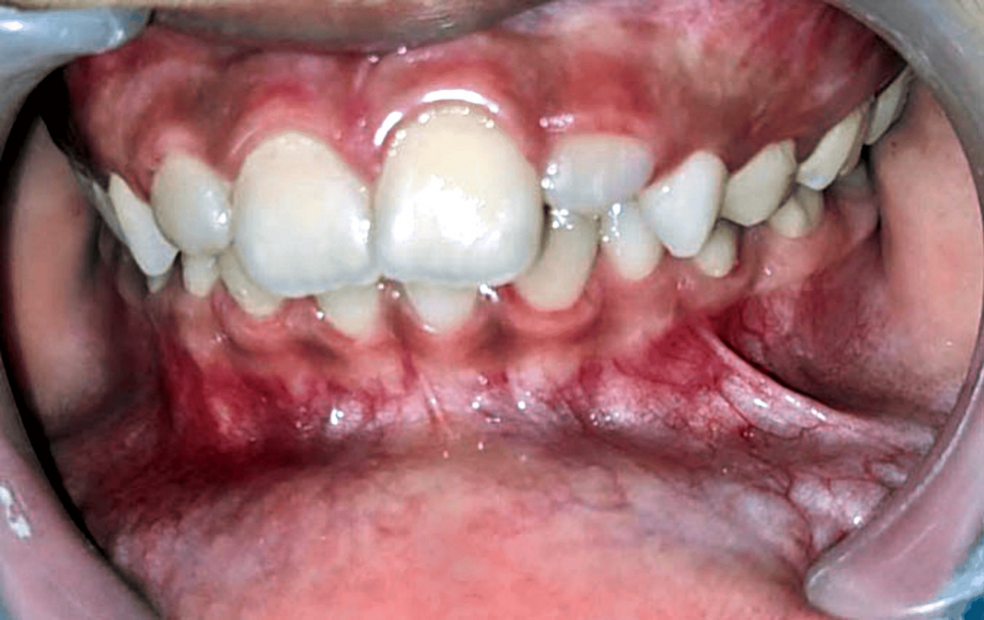
Follow-up image showing complete healing.

The child was counseled to change the habit and was kept on periodic follow-ups to assess the healing and look for any reoccurrence. Follow-up was done after three weeks and healing had occurred uneventfully (Figure 6).

Case two

A six-year-old patient presented with complaints of swelling on the left side of the lower lip. The child had a history of trauma to his lower lip while playing three months back. After the trauma, swelling appeared on the site, which was smaller initially but had increased in size. The patient did not have any associated pain or a history of pus or blood discharge. No significant medical or dental history was given.

**Figure 7:**
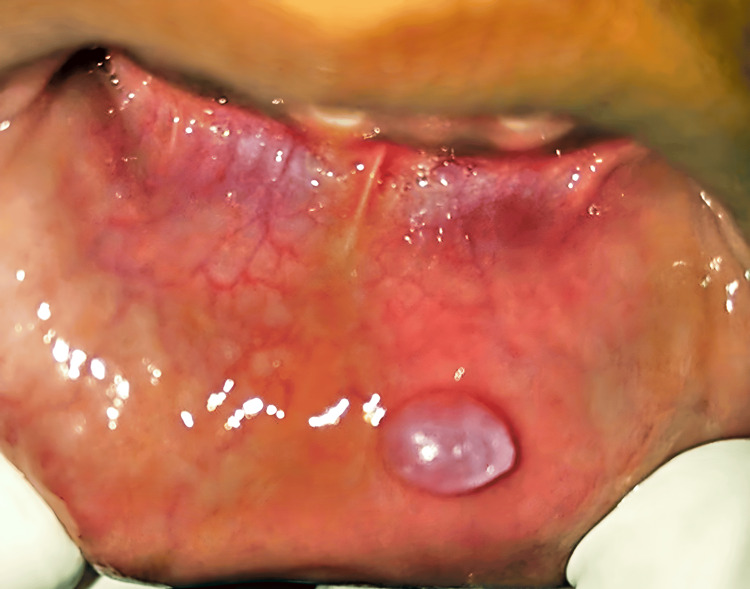
Examination of the lip showing a mass on the left side.

On examination, a pinkish-blue, oval swelling was noted on the left side of the lower lip about 31 and 32 which was roughly 0.5 x 0.4 cm in diameter (Figure 7).

**Figure 8:**
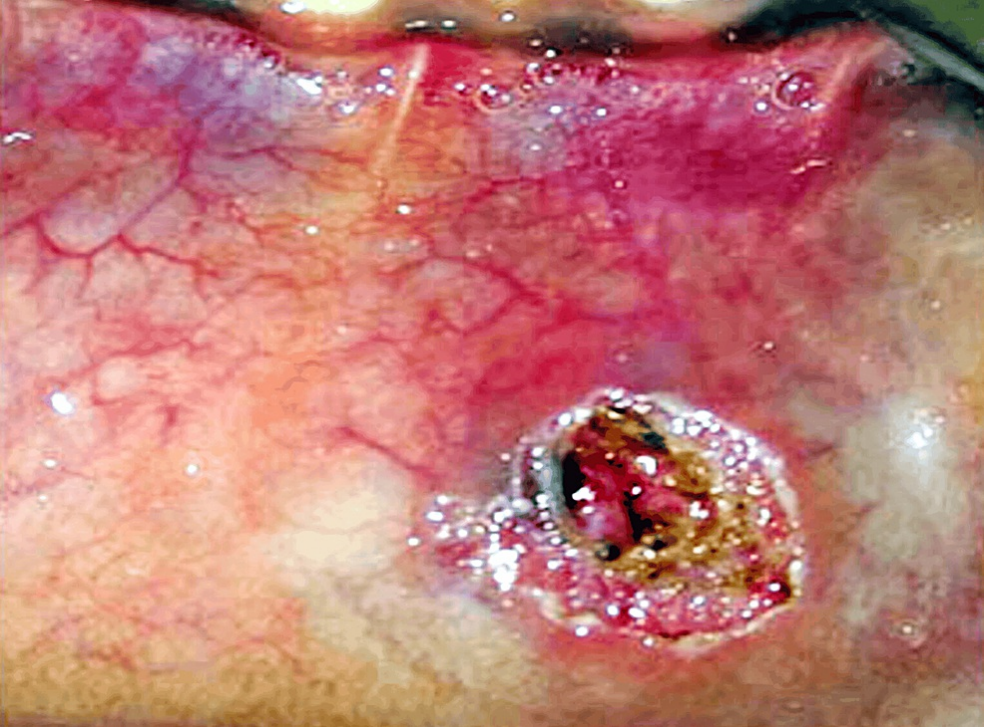
Immediate postoperative image showing LASER excision.

On palpation, the swelling was fluctuant and non-tender. Moreover, no rise in temperature was noted. Based on the examination, the swelling was diagnosed provisionally as mucocele, for which LASER excision was planned (Figure 8).

**Figure 9:**
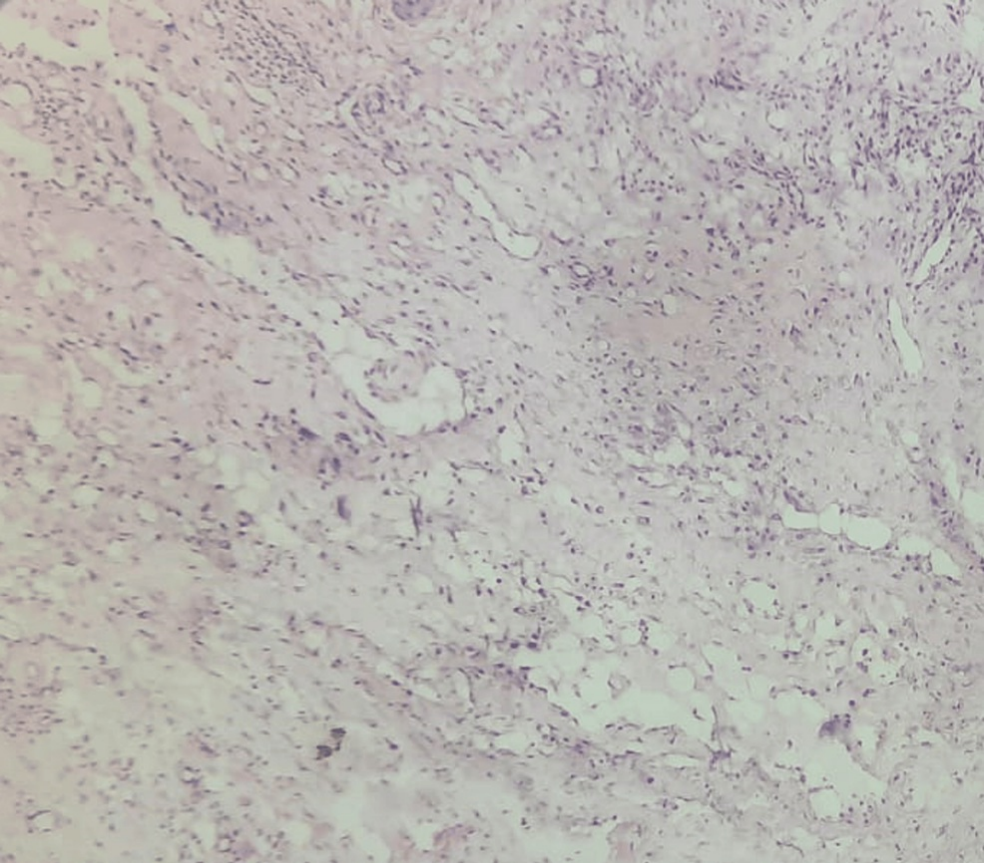
Histopathological image showing mucus extravasation into the tissue stained with hematoxylin and eosin at 10× magnification showing mucous pooled area surrounded by inflammatory cells with scarce amounts of connective tissue fibers.

Using a 940 nm diode LASER in contact mode, the mucocele was removed and sent for histological investigation, which confirmed the provisional diagnosis (Figure 9).

**Figure 10:**
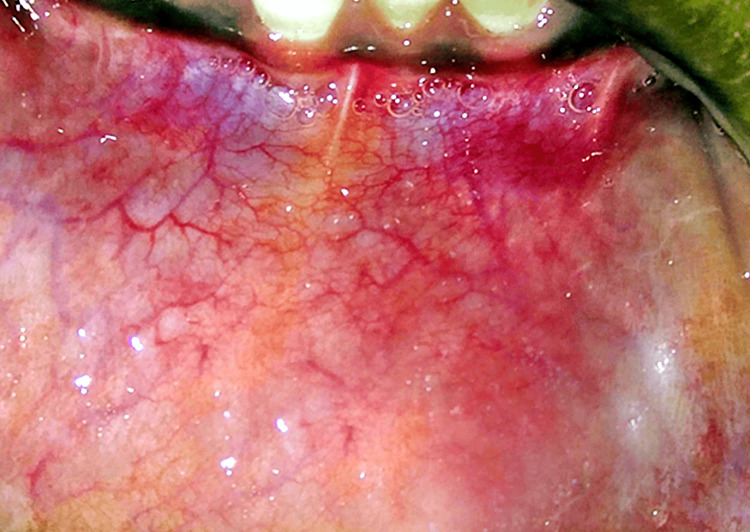
Follow-up image showing complete healing.

The patient was kept on periodic follow-ups to evaluate the healing process. After three weeks, complete healing had occurred (Figure 10).

## Discussion

Pediatric patients are susceptible to a wide range of oral conditions, and one uncommon but noteworthy condition is traumatic fibroma of the lower lip [1]. Traumatic fibroma is a non-cancerous, reactive lesion that can develop anywhere in the oral cavity, including the lower lip [3-5]. Fibroma can develop from different types of localized trauma or persistent irritation, in addition to self-biting. In pediatric patients, common factors contributing to its development include chronic lip biting, foreign body reactions or dental appliances that continuously irritate the lip tissues, dental malocclusions wherein the poorly aligned teeth can create unnatural forces on the lips and cheeks during mastication thereby increasing the risk of lip trauma, thumb or finger sucking habits resulting in chronic irritation of the lower lip leading to fibrous tissue formation, and accidental injuries such as fall and friction against braces or orthodontic appliances. The constant irritation and friction lead to excessive proliferation of fibrous connective tissue at the affected site, resulting in the formation of a firm, painless lump [1,3,6].

Mucocele, on the other hand, also known as a mucous-filled cyst, is another benign lesion that develops from the blockage or rupture of a minor salivary gland duct, leading to the accumulation of liquid or mucoid material in the surrounding within the submucosal tissues [7,8]. This is caused by lip biting, accidental injuries, or even repetitive trauma from dental appliances such as orthodontic brackets or space maintainers with sharp points. The spilling of mucin into connective tissue causes an inflammatory reaction, which results in the creation of a connective tissue wall formed of granulation tissue to restrict the location of mucin spillage. When mucus pooling occurs beyond the constriction of the salivary duct, it is not bordered by epithelium and is thus classified as a pseudocyst [7-10]. Unlike traumatic fibroma, mucocele is more prevalent in older children, adolescents, and young adults [2,6,7].

Traumatic fibromas commonly present as solitary, slow-growing, well-circumscribed, firm, and painless nodules on the lower lip; these lesions may have a smooth or slightly irregular surface and frequently resemble a small tumor or growth. The color might range from pink to normal mucosal hue. In certain instances, such as trauma or irritation, the lump may become inflamed or traumatized, resulting in sporadic bleeding. It may also grow significantly, causing discomfort while speaking, eating, or other activities. Due to the location on the lower lip, patients may also be concerned about the aesthetic appearance of the affected area [1-4]. The oral manifestation of mucocele or mucus cyst is a soft, fluid-filled protrusion on the lower lip that is bluish or transparent. Depending on the amount of saliva accumulated, mucocele may fluctuate in size. Contrary to traumatic fibromas, mucoceles are more likely to rupture spontaneously, leading to a brief reduction in size and then re-accumulation of saliva. If infected or inflamed, it may hurt [8-11].

A comprehensive clinical examination is often required to diagnose a traumatic fibroma and mucocele of the lower lip which includes an assessment of the size, shape, and texture of the mass along with establishing a thorough medical and dental history. The examination takes into account the size, form, and texture of the mass. A biopsy can help confirm the nature of the lesion and rule out other conditions, such as a malignancy. On histopathologic examination of mucocele, extravasation of mucus into neighboring tissues, loss of ductal epithelium, and mucus infiltration of connective tissue encircled by chronic inflammatory cells and covered by a para-keratinized stratified squamous epithelium can be seen. On the other hand, fibroma histologically exhibits a connective tissue defect and is distinguished by fibroblast proliferation and collagen fiber deposition in condensed, small beams [3,6].

Depending on the size and symptoms of the lesion, traumatic fibroma in pediatric children may require a different approach to treatment. Small asymptomatic fibromas can be monitored to resolve spontaneously. However, the following treatment alternatives may be taken into account if the fibroma causes discomfort, interferes with oral functions, or raises cosmetic issues: excision surgery wherein the fibroma is surgically excised under local anesthesia. To avoid recurrence, care should be taken to ensure that the nodule is completely removed together with a margin of healthy tissue. Laser ablation or cryotherapy, being less invasive, can also successfully eliminate the fibroma and hasten the healing process. In cases where fibroma is linked to habits, such as thumb sucking or lip biting, behavioral modification techniques must be implemented to break the habit and stop further trauma [5,6]. Mucocele can be treated similarly to fibroma by surgical excision, marsupialization, or LASER treatment. The main goal is to remove the mucocele and the afflicted gland to prevent recurrence. However, it is crucial to address the contributing factors to reduce the likelihood of future occurrences [11,12]. After the surgical excision, pediatric patients may experience mild discomfort or swelling for a few days. Proper wound care and following the postoperative instructions are crucial for a smooth recovery [6]. In this case report, both cases were treated with LASER, which resulted in less post-treatment discomfort. Moreover, both patients accepted the mode of treatment quickly. Conventional methods could have been chosen for the management, and long-term follow-up could have been done.

Clinical and histopathological examinations play a pivotal role in diagnosing these conditions along with differentiating them from one another. This paper not only gives a thorough understanding of the lesions, mucocele, and traumatic fibroma but also sheds light on their distinguishing features along with microscopic features. Both lesions could be troublesome for the child and hamper speech, lip closure, etc., and can lead to an unaesthetic appearance of the child; therefore, treatment of these lesions is necessary.

## Conclusions

Both traumatic fibromas and mucoceles are benign oral lesions that typically affect the lips. Although both tumors appear as nodules on the lower lip, their etiology and diagnostic techniques are very different. As a result, a comprehensive understanding of these variations is required for accurate diagnosis and treatment. Correct diagnosis and proper care are critical in attaining the best possible outcomes for those suffering from these benign oral disorders. Regular dental check-ups and prompt action can also help avert complications and promote general oral health and well-being.
